# Caveolin-1-Mediated Apolipoprotein A-I Membrane Binding Sites Are Not Required for Cholesterol Efflux

**DOI:** 10.1371/journal.pone.0023353

**Published:** 2011-08-12

**Authors:** Soazig Le Lay, Macarena Rodriguez, Wendy Jessup, Carles Rentero, Qiong Li, Siân Cartland, Thomas Grewal, Katharina Gaus

**Affiliations:** 1 Centre de Recherche des Cordeliers, INSERM, U872, Paris, France; 2 Centre for Vascular Research, University of New South Wales, Sydney, Australia; 3 Faculty of Pharmacy, University of Sydney, Sydney, Australia; Institut Curie, France

## Abstract

Caveolin-1 (Cav1), a structural protein required for the formation of invaginated membrane domains known as caveolae, has been implicated in cholesterol trafficking and homeostasis. Here we investigated the contribution of Cav1 to apolipoprotein A-I (apoA-I) cell surface binding and intracellular processing using mouse embryonic fibroblasts (MEFs) derived from wild type (WT) or Cav1-deficient (Cav1^−/−^) animals. We found that cells expressing Cav1 have 2.6-fold more apoA-I binding sites than Cav1^−/−^ cells although these additional binding sites are not associated with detergent-free lipid rafts. Further, Cav1-mediated binding targets apoA-I for internalization and degradation and these processes are not correlated to cholesterol efflux. Despite lower apoA-I binding, cholesterol efflux from Cav1^−/−^ MEFs is 1.7-fold higher than from WT MEFs. Stimulation of ABCA1 expression with an LXR agonist enhances cholesterol efflux from both WT and Cav1^−/−^ cells without increasing apoA-I surface binding or affecting apoA-I processing. Our results indicate that there are at least two independent lipid binding sites for apoA-I; Cav1-mediated apoA-I surface binding and uptake is not linked to cholesterol efflux, indicating that membrane domains other than caveolae regulate ABCA1-mediated cholesterol efflux.

## Introduction

Expression of caveolin-1 (Cav1) and cavin proteins in mammalian cells drives the formation of small (50–100 nm) invaginations of the plasma membrane known as caveolae [1], [2], [3]. Caveolae are regarded as a subtype of a group of specialized membrane microdomains, i.e. lipid rafts [4] that are characterized by their enrichment in cholesterol and sphingolipids [5], [6]. Both the expression of Cav1 and the formation of caveolae are dependent on cellular cholesterol levels [7]. Cav1 mutations in patients are linked to lipid disorders [8] and Cav1 itself is a high-affinity cholesterol binding protein [9]. Cav1 has been implicated in controlling the intracellular balance between free and esterified cholesterol [10] and interacting with many lipid transporters [11]. Studies in which Cav1 expression is increased or knocked out indicate that Cav1 regulates storage of lipids in cytoplasmic lipid droplets in adipocytes and hepatocytes [12], [13], [14]. It has also been suggested that Cav1 promotes the transport of newly synthesized cholesterol from the endoplasmic reticulum to outer leaflet of the plasma membrane, where it is accessible to extracellular cholesterol oxidase [15], [16].

Cholesterol export from the plasma membrane to extracellular acceptors is the first step in the ‘reverse cholesterol transport’ pathway, which delivers excess cholesterol from peripheral tissues back to the liver. Cholesterol efflux is tightly controlled, and the pathways involved depend on the nature of the extracellular acceptor [17]. Cholesterol efflux to apolipoprotein A-I (apoA-I), the major protein component of high-density lipoproteins (HDL), utilizes the ATP-binding cassette transporter A1 (ABCA1), which is probably not associated with lipid rafts [18] but may alter cholesterol distribution within the plasma membrane [19], [20], [21], [22]. Efflux to HDL is mediated by other transporters such as scavenger receptor class B type I (SR-BI) and ABCG1 [20], [23], [24], [25]. Treatments that disrupt lipid raft structure have little effect on HDL-dependent cholesterol export [26] but raft integrity may be required for HDL-induced cholesterol ester exchange [27].

There are inconsistent reports regarding the role of Cav1 in cholesterol export. Fu *et al.* transiently increased Cav1 in hepatic cells and found enhanced cholesterol export to apoA-I and HDL-containing plasma [28] (later confirmed in [29]), which is consistent with the previous finding that apoA-I induces cholesterol trafficking to caveolae [30]. Reducing expression of Cav1 in human monocyte-derived macrophages also reduced cholesterol efflux to apoA-I and HDL [31]. In contrast, down-regulation of Cav1 in NIH3T3 fibroblast increased cholesterol efflux to HDL [32] contradicting earlier results in human skin fibroblasts [33]. Over-expression of Cav1 in mouse macrophage cell lines had no effect on HDL-mediated efflux [34]. In embryonic fibroblasts and peritoneal macrophages from Cav1-deficient mice (Cav1^−/−^), cholesterol export to HDL was not different to that from the wild-type (WT) cells [10]. Those differences may be attributed to differential effects in different cell types and the effect Cav1 expression has on other cholesterol transporters. For example, Cav1 expression inhibits SR-BI-mediated cholesterol influx [34] although this was not the case in HEK-293T cells [35]; increases cholesterol uptake in macrophages [36] and hepatic cells [29]; and had no effect uptake and export in Fischer rat thyroid cells and HEK 293 cells [37]. The effect of Cav1 expression on ABC transporter has been reported for endothelial cells, in which Cav1 expression correlates with ABCA1 expression and thus modulates cholesterol efflux [38], [39].

To add to the complications, there is an ongoing debate whether apoA-I internalization and recycling is required for apoA-I-mediated efflux or whether apoA-I interactions with lipid domains on the cell surface are sufficient. An earlier report found that a substantial proportion of apoA-I was endocytosed and re-secreted from mouse macrophages [40]. This has been recently confirmed suggesting that in mouse macrophages ABCA1-mediated apoA-I, but not HDL uptake is required for cholesterol efflux [41]. Similarly, Hassan et al. suggest that apoA-I re-secretion is mainly responsible for cholesterol export from BHK cells that stably express ABCA1 [42]. In contrast, two recent papers found that apoA-I retroendocytosis is not sufficient for cholesterol efflux from mouse macrophages [43] and ABCA1-expressing BHK cells [44].

Given this controversy and that earlier papers studied cholesterol efflux independently from apoA-I binding and processing, we re-investigated how Cav1 expression affects apoA-I binding to the plasma membrane, apoA-I internalization and cholesterol efflux. We used wild-type (WT) and Cav1^−/−^ mouse embryonic fibroblasts (MEFs) because the genetic deletion of Cav1 ensures that Cav1^−/−^ MEFs have no detectable caveolae [45] but comparable levels of SR-BI, ABCA1 and ABCG1, as found here. We induced the expression of ABCA1 in these cells with a potent LXR agonist, T0901317. We found a higher number of apoA-I binding sites on WT cells than Cav1^−/−^ MEFs, which correlated with enhanced apoA-I internalization and degradation. However, Cav1-mediated internalization was not required for cholesterol efflux in this cell type. In summary, our results indicate that surface binding outside caveolae is sufficient to regulate ABCA1-mediated cholesterol efflux from fibroblast suggesting that membrane domains other than caveolae have an important role in cholesterol export.

## Materials and Methods

### Cells and Reagents

Mouse embryonic fibroblasts (MEFs) were prepared from 13.5 p.c. embryos obtained by homozygous crossings of Cav1^−/−^ mice or WT mice (approved by the Regional Ethics Committee for Animal experiment, N°3 of Ile de France, Paris; Approval ID : p3/2008/013) [45]. MEF cells were immortalized by continuous passages until growth rates in culture resumed the rapid rates seen in early passages. MEFs were cultured in Dulbecco's modified Eagle's medium (DMEM) supplemented with 10% (v/v) FCS, 2 mM L-glutamine, 100 units/L penicillin, and 100 µg/L streptomycin at 37°C in 5% CO_2_. Where indicated, cells were treated with the LXR ligand T0901317 (1 µM) for 18 h. Bovine serum albumin (BSA, essentially fatty acid free), Dulbecco's phosphate buffered saline (PBS), chloramphenicol, cholesterol and cholestenone were purchased from Sigma. [^3^H]-cholesterol (48.0 Ci/mmol) and [^125^I]-iodine were obtained from Amersham. ApoA-I was delipidated and purified as described previously [46].

### ApoA-I binding measurements

#### ApoA-I labeling

Iodine incorporation into apoA-I was carried out as described previously [47]. Briefly, ^125^Iodine (10 µCi; Amersham) was incubated with one IODO bead (Pierce) for 2 min before apoA-I (1–3 mg) was added. After 1 min, free iodine was separated from [^125^I]-apoA-I by gel filtration (PD10; Pharmacia). Typically, a specific activity of 500 dpm/ng apoA-I was achieved with <3% free iodine contamination.

#### ApoA-I binding at 4°C

For binding experiments, cells were place on ice and incubated with [^125^I]-apoA-I (0–75 µg/mL) in DMEM for 90 min at 4°C. Non-specific binding, typically ∼20% of specific binding independent of cell type, was measured in the presence of a 10-fold excess of unlabelled apoA-I [47]. Specific binding was calculated from the difference between total and non-specific binding. At the end of the incubation, cells were washed twice to remove unbound apoA-I and cells were lysed in 0.2 M NaOH and counted for [^125^I] radioactivity.

#### Binding coefficients

To obtain values for maximal apoA-I binding (B_max_, ng/mg) and the apoA-I concentration at which half of the maximum binding was achieved (K_D_, µg/mL), apoA-I calibration curves were fitted to
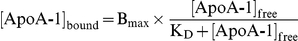
using a non-linear fitting algorithm (Microsoft Excel).

#### ApoA-I binding to membrane domains

To determine apoA-I binding to membrane domains, cells were first incubated with [^125^I]-apoA-I at 4°C and then washed and fractionated by sonication and placed on a 5–45% sucrose gradient as described below. Fractions were analyzed for [^125^I]-apoA-I. For controls, cells pre-labeled with [^3^H]-cholesterol without [^125^I]-apoA-I and [^125^I]-apoA-I without cells were fractionated. 95% of unbound apoA-I was found in the heaviest fraction of the non-detergent raft gradient und these controls were used to calculate the percentage of bound apoA-I in each fraction as previously described [47].

#### Non-detergent raft isolation

Whole cell homogenates (total volume 1.2 mL) were sonicated for 4×30 s with a 3 mm titanium probe (frequency 23 kHz, amplitude 30 microns) and cooled on ice between bursts [48]. Samples were then adjusted to a final concentration of 45% (w/v) sucrose by mixing with an equal volume of 90% (w/v) sucrose in MBS (25 mM MES pH 6.5, 150 mM NaCl). 2.0 mL of the mixture was overlaid with 2.5 mL of 35% (w/v) sucrose, 2.5 mL of 30% (w/v) sucrose, 2.5 mL of 25% (w/v) sucrose and 2.5 mL of 5% (w/v) sucrose (all in MBS) [48]. The sucrose gradient was spun at 40,000 RPM in a Beckman SW41 rotor (200,000 g) for 16 h. Twelve fractions of 1.0 mL were collected from the top.

#### ApoA-I binding and processing at 37°C

WT and Cav1^−/−^ MEFs were incubated with 25 µg/mL ^125^I-apoA-I ±10-fold unlabeled apoA-I at 37°C for 15 min, 30 min, 1 h or 2 h. Specific apoA-I binding was calculated as described above. Non-specific binding in the presence of 10-fold excess of unlabeled apoA-I was similar in WT and Cav1^−/−^ MEFs and comparable to binding experiments at 4°C. At the indicated times, the media were collected, detached cells removed and degraded apoA-I in the media determined as TCA-soluble ^125^I. Briefly, 0.25 mL media was mixed with 0.5 mL of ice-cold 50% TCA (Sigma) and 125 µL of 0.7 M AgNO_3_ (Sigma). The mixture was spun at 200 g for 15 min and the supernatant collected and counted for degraded ^125^I-apoA-I. After the media was removed, cells were washed twice with ice-cold PBS and incubated with 0.5 mg/mL trypsin (Sigma) for 30 min at 4°C on ice. Supernatants were collected, detached cells removed and ^125^I-apoA-I counted to determine trypsin-sensitive e.g. surface-bound apoA-I. The remaining cells were lysed, protein levels determined and trypsin-resistant ^125^I-apoA-I counted to quantify internalized apoA-I. At each time point and in each of the three compartments (surface-bound, internalized and degraded apoA-I), non-specific apoA-I was similar in both cell types and was subtracted to determine specific surface-bound, internalized and degraded ^125^I-apoA-I. It should be noted that the trypsin treatment that defined surface-bound apoA-I is only 89±3% efficient (as quantified by apoA-I binding at 4°C) so that the internalized (trypsin-resistant) apoA-I pool is slightly overestimated.

### Immunoblotting

Cell lysates and plasma membranes isolated by subcellular fractionation were prepared as previously described [47]. Equal amounts of protein were run on 12.5% SDS/PAGE gels, blotted and probed with various antibodies described in the figure legends. Primary antibody detections were performed using appropriate peroxidase-conjugated secondary IgGs and visualized using enhanced chemiluminescence by exposure to Kodak autoradiographic film. The polyclonal caveolin-1 antibody was from Santa Cruz biotechnology. ABCA1 and SR-BI antibodies were from Novus.

### Sterol analysis

Cell lysates, plasma membrane and NDR were extracted as described previously [46] and cholesterol and cholesterol esters were analysed by reverse-phase HPLC.

### Cholesterol and phospholipid efflux

To measure cholesterol efflux, cells were incubated for 24 h with [1a,2a(n)-^3^H]cholesterol with and without LXR ligand T0901317 (1 µM). To measure phospholipid efflux, cells were labeled with [methyl-^3^H] choline chloride (Amersham). The cells were washed twice with PBS followed by 90 min equilibration in serum-free DMEM containing 1 mg/ml BSA (fatty acid-free). Cholesterol efflux was measured in DMEM containing 1 mg/mL BSA and with or without 10 µg/mL apoA-I or HDL_2_ by determining [^3^H]-cholesterol in aliquots (100 µL) of media taken at intervals [49], [50]. Phospholipid efflux was determined after a 24 h efflux. At the end of the experiments cells were washed twice in ice-cold PBS and lysed in 1 mL NaOH (0.2 M, Sigma). Residual radioactivity was determined in the cell lysate. Efflux is expressed as a percentage of the total counts in the system.

## Results

### Characterization of WT and Cav1^−/−^ MEFs

We started our investigations with a characterization of WT and Cav1^−/−^ MEFs. Cav1 expression is essential for the formation of caveolae, and cells from Cav1^−/−^ mice show a complete lack of morphologically identifiable caveolae [2], [45]. Cav1^−/−^ MEFs contained no detectable levels of Cav1 while Cav2 levels in Cav1^−/−^ MEFs were severely reduced (12.8±10.6%) compared to WT MEFs [51].

**Figure 1:**
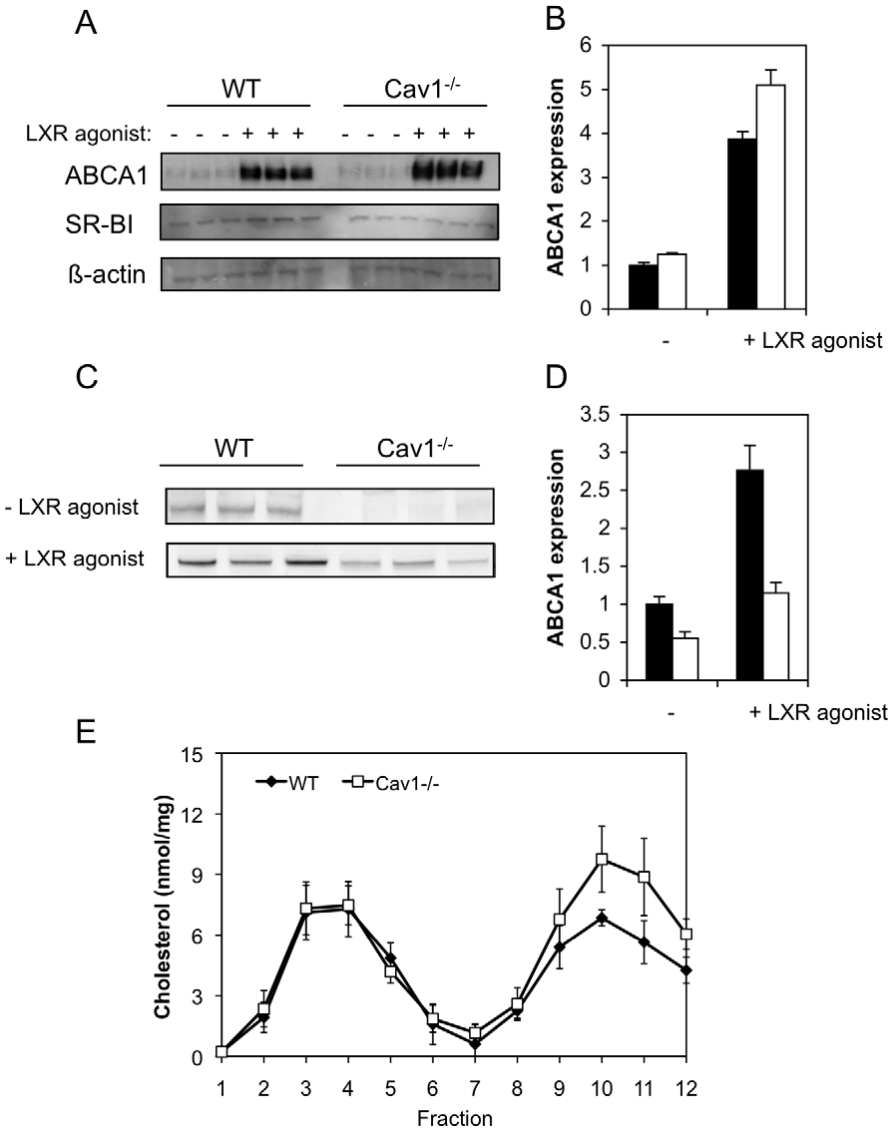
Characterization of WT and Cav1^**−/−**^ MEFs. **A.** Protein expression of ABCA1 and SR-BI of WT and Cav1^−/−^ MEF with and without LXR agonist T0901317 treatment. **B.** Quantification of ABCA1 protein expression of Cav1^−/−^ MEFs (hollow bars) relative to WT MEFs (filled bars) without and with LXR agonist T0901317 treatment. **C.** ABCA1 expression in the plasma membrane with and without LXR agonist T0901317 treatment. **D.** Relative ABCA1 cell surface expression in WT MEFs (filled bars) and Cav1^−/−^ MEFs (hollow bars) without and with LXR agonist T0901317 treatment. **E.** Cholesterol content in NDR and non-raft membranes in WT and Cav1^−/−^ MEFs. Light and heavy membranes were prepared from whole cell homogenate by sonication and separation on a 5–45% sucrose gradient as described in *Methods*. Twelve fractions are collected from the top of the gradient. The distribution of cholesterol across the gradient was determined by fractionating WT (closed diamond) and Cav1^−/−^ MEFs (open squares) with incorporated ^3^H-cholesterol. The data show average ± standard deviation of three independent experiments.

ApoA-I mediated-cholesterol export critically depends on the expression of ABCA1, a transporter that associates with non-raft domains [47]. Under basal conditions, WT and Cav1^−/−^ MEFs expressed comparable levels of ABCA1 protein (Fig. 1A and B) and mRNA (data not shown). To increase ABCA1 expression, we treated cells with the specific LXR agonist T0901317. ABCA1 protein expression was increased ∼4-fold in both cell types by the LXR agonist and was slightly (∼1.2-fold) higher in Cav1^−/−^ than WT MEFs (Fig. 1B). In contrast, SR-BI protein (Fig. 1A) and mRNA levels (not shown) were similar between the two cell types and were not affected by treatment with the LXR agonist. ABCG1 levels were also not significantly different, as judged by RT-PCR (data not shown). We also assessed ABCA1 expression in the plasma membrane (Fig. 1C). In response to LXR agonist treatment, both cell types increased ABCA1 surface expression ∼2-fold. However, despite higher cellular ABCA1 levels, Cav1^−/−^ MEFs had respectively 1.8-fold and 2.4-fold less ABCA1 at the cell surface than WT MEFs in the absence and presence of the LXR agonist (Fig. 1D).

Because caveolae are regarded as a subtype of lipid rafts [4] and are enriched in cholesterol, we examined whether lipid raft abundance is different in these two cell types. Lipid rafts including caveolae can be isolated based as detergent-resistant membranes (DRMs) or as a light membrane sub-fraction obtained after mechanical disruption of cell membranes [48]. In all cell types tested, Cav1 is predominantly associated with DRMs or light membrane fractions of membranes [52]. Here, we compared the cholesterol content of non-detergent raft domains (NDR) between WT and Cav1^−/−^ MEFs (Fig. 1E) that were prepared from cell homogenates by sonication followed by separation on a 5–45% sucrose density gradient. We previously reported that membrane-associated cholesterol levels are similar in WT and Cav1^−/−^ MEFs [51] and that ABCA1 is exclusively found in non-raft factions on this gradient [47]. Lipid raft-favoring proteins Cav1 and flotillin-1 partitioned predominantly into fractions 2–5, while the non-raft protein transferrin receptor was exclusively found in fractions 8–12 (data not shown) [48]. WT MEFs contained 43.3±2.0% (or 21.2±2.1 nmol/mg cell protein) of cholesterol in NDR compared to 35.4±5.3% (21.3±2.2 nmol/mg) in Cav1^−/−^ MEFs (mean ± s.d. of 3 separate experiments). Although the proportion of cholesterol in NDR was consistently lower in Cav1^−/−^ MEFs, the difference in proportional distribution between WT and Cav1^−/−^ NDR cholesterol was not statistically significant (P>0.05). A previous study indicated that the distribution of proteins normally associated with lipid rafts is unaffected by changes in Cav1 expression [53]. Together with the data presented here, it can be speculated that either total lipid raft domains (caveolae+non-caveolae rafts) are not dependent on Cav1 expression, or that caveolae represent only a small proportion of total raft cholesterol.

### ApoA-I binding to WT and Cav1^−/−^ MEFs

Caveolae are the preferred binding sites of raftophilic toxins and apoA-I binds directly to protein-free lipid bilayers. Thus it is possible that caveolae are specific apoA-I binding sites. Therefore we compared apoA-I surface binding to Cav1^−/−^ MEFs with binding to WT cells.

**Figure 2:**
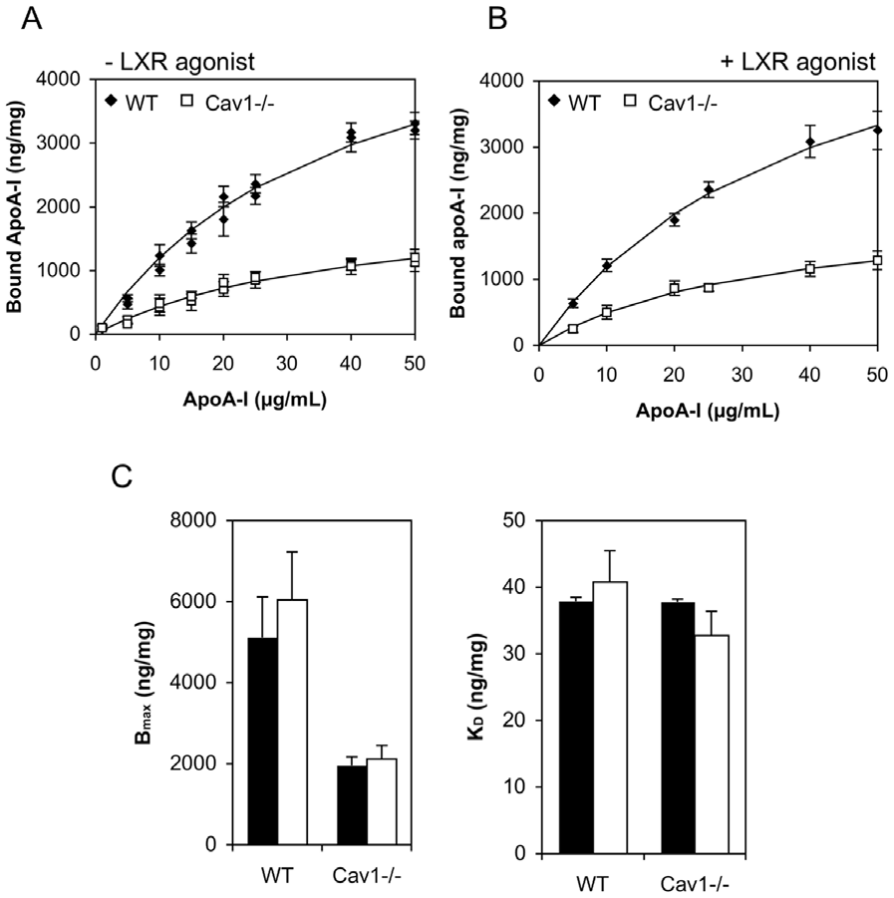
ApoA-I binding to WT and Cav1^**−/−**^ MEFs at 4°C. **A.–B.** ^125^Iodine-labeled apoA-I (0–50 µg/mL) was bound to WT (solid diamonds) and Cav1^−/−^ MEFs (open squares) at 4°C on ice. Prior to exposure to apoA-I, cells in B, but not A, were treatment with the LXR agonist T0901317. Specific binding was determined by competition with 10-fold excess of unlabeled apoA-I. Specifically bound apoA-I was normalized to cell protein. The data (symbols) are a representative of independent experiments each performed in triplicate cell cultures. Solid lines represent best fits as described in *Methods*. **C.** The number of maximum binding sites, B_max_ and the binding affinity parameter K_D_ (− LXR agonist shown in filled bars, + LXR agonist hollow bars) obtained from the fit are shown as average ± range of two independent experiments each performed in triplicates. No difference (P>0.05) was found ± LXR agonist treatment.

To measure binding, ^125^I-labelled apoA-I was bound to cells at 4°C. Non-specific binding was determined in the presence of 10-fold excess of unlabelled apoA-I (similar in both cell types, data not shown) and subtracted from total bound apoA-I to determine specific apoA-I binding (Fig. 2). Specific binding was fitted to Langmuir equation (Fig. 2A and B) to obtain the binding affinity (K_D_) and number of binding sites (B_max_). While the binding affinity for apoA-I was similar for both WT and Cav1^−/−^ MEFs, WT cells displayed ∼2.6-fold more apoA-I binding sites compared to Cav1^−/−^ MEFs (Fig. 2C).

Increasing ABCA1 expression levels by pretreatment with an LXR agonist had no effect on apoA-I binding affinity (K_D_) and number of binding sites (B_max_) in either cell type (Fig. 2B and C). Even when ABCA1 surface expression is taken into account (Fig. 1D), our data indicates that Cav1 expression, but not ABCA1 expression, increases apoA-I surface binding sites in MEFs: Cav1^−/−^ MEFs treated with the LXR agonist had similar ABCA1 expression in the plasma membrane than untreated WT MEFs, yet their apoA-I binding capacity is vastly different. In previous reports, increased expression of ABCA1, either by stimulation or ectopic expression increased apoA-I bindings sites in the mouse macrophage line J774 [54] and BHK cells [42], [55]. It is noteworthy that neither of these cell types expresses detectable levels of caveolin or caveolae [56], [57] so that it is possible that apoA-I binding in the complete absence of both ABCA1 and Cav1, which is the basal condition in these studies, is non-specific. This is not the case in Cav1^−/−^ MEFs, which not only express ABCA1 (Fig. 1A–D) but to which apoA-I binds with a similar high affinity than to WT MEFs (k_D_ in Fig. 2C).

Vedhachalam et al suggest that ABCA1-expressing cells have two types of high affinity binding sites: apoA-I/ABCA1 interaction that account for only ∼10% of bound apoA-I have a low capacity (low B_max_) while apoA-I/lipid domain interactions constitute high capacity binding sites [54]. It is thus possible that caveolae in WT MEFs are high-affinity, high capacity apoA-I binding sites, while the high-affinity, low capacity binding sites on Cav1^−/−^ MEFs are ABCA1-mediated. A 2-fold increase in ABCA1 surface expression under LXR agonist treatment did increase B_max_ by 18% and 9% in WT and Cav1^−/−^ MEFs, respectively, but this increase was not significant in either cell type given that the standard error for B_max_ was >10%. Hence, the low capacity of ABCA1-mediated apoA-I binding site may explain why a 2-fold increase in ABCA1 surface expression did not significantly increase total apoA-I binding.

### ApoA-I binding to Cav1-enriched membrane domains

We have previously shown that apoA-I binds predominantly to non-raft domains on the plasma membrane of macrophages, although a small, but significant proportion of apoA-I binds to cholesterol-rich non-detergent rafts (NDR). Furthermore, we demonstrated that apoA-I binding to the NDR was essential for stimulation of cholesterol efflux [47].

**Figure 3:**
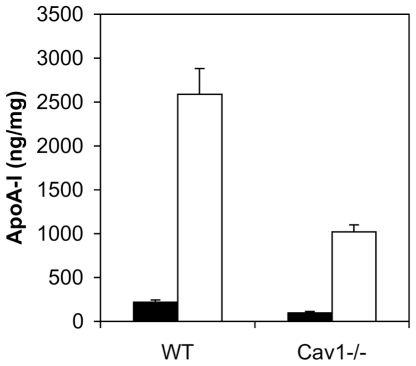
ApoA-I binding to NDR and non-raft membranes in WT and Cav1^**−/−**^ MEFs. ApoA-I binding to light (fractions 2–5, filled bars) and heavy (fractions 8–12, hollow bars) membranes was quantified by scintillation counting. ApoA-I (50 µg/mL) was bound at 4°C on ice and MEFs were fractionated into NDR and non-raft membranes as described for Fig. 1E. Data are average ± standard deviation of five experiments.

We next compared the proportion of apoA-I surface binding to membrane domains that are enriched in caveolae (Fig. 3). To determine apoA-I binding to raft and non-raft domains, we fragmented cells to which ^125^I-labelled apoA-I was first bound at 4°C. ^125^I-labelled apoA-I that detached from membranes (13.7±5.6% of apoA-I bound to either cell type) appeared as a pellet at the bottom of the gradient [47] and was not included in the data in Figure 3. Similar to Figure 2, we found a 2.5-fold reduction of apoA-I binding to Cav1^−/−^ MEFs compared to WT MEFs. The reduction in binding is particularly obvious in non-raft domains: the vast proportion of the additional binding sites associated with expression of Cav1 was not raft-associated because they partitioned into non-raft domains in the isolation procedure. However, when apoA-I in NDR is expressed as a proportion of bound apoA-I, we found no difference between WT MEFs (7.3±1.3% of bound apoA-I in NDR) and Cav1^−/−^ MEFs (8.2±0.6% of bound apoA-I in NDR) and consequently no difference in proportional apoA-I binding to non-rafts between WT (85.3±3.2%) and Cav1^−/−^ MEFs (86.3±0.4%). Hence the expression of Cav1 had no significant effects on cholesterol levels in NDR (Fig. 1E), nor on the relative distribution of apoA-I between raft and non-raft membrane domains. In summary, we made the surprising discovery that Cav1 expression in MEF induce apoA-I binding sites that have the biochemical characteristics of non-raft domains.

### ApoA-I processing

Both caveolae and non-caveolar rafts can act as entry portals for bacterial toxins, viruses and plasma proteins, as reviewed in reference [1]. Cav1^−/−^ fibroblasts have impaired uptake of albumin (but not transferrin receptor [2]) and reduced internalization of raft marker GM1 whose uptake in wildtype cells is initiated when integrin are dis-engaged and cells are detached from the substratum [58], [59]. It has been suggested that internalization of apoA-I is important for stimulation of cholesterol export [40], [60], [61] but it is not known if apoA-I endocytosis involves caveolae and whether caveolar uptake is required for cholesterol export.

**Figure 4:**
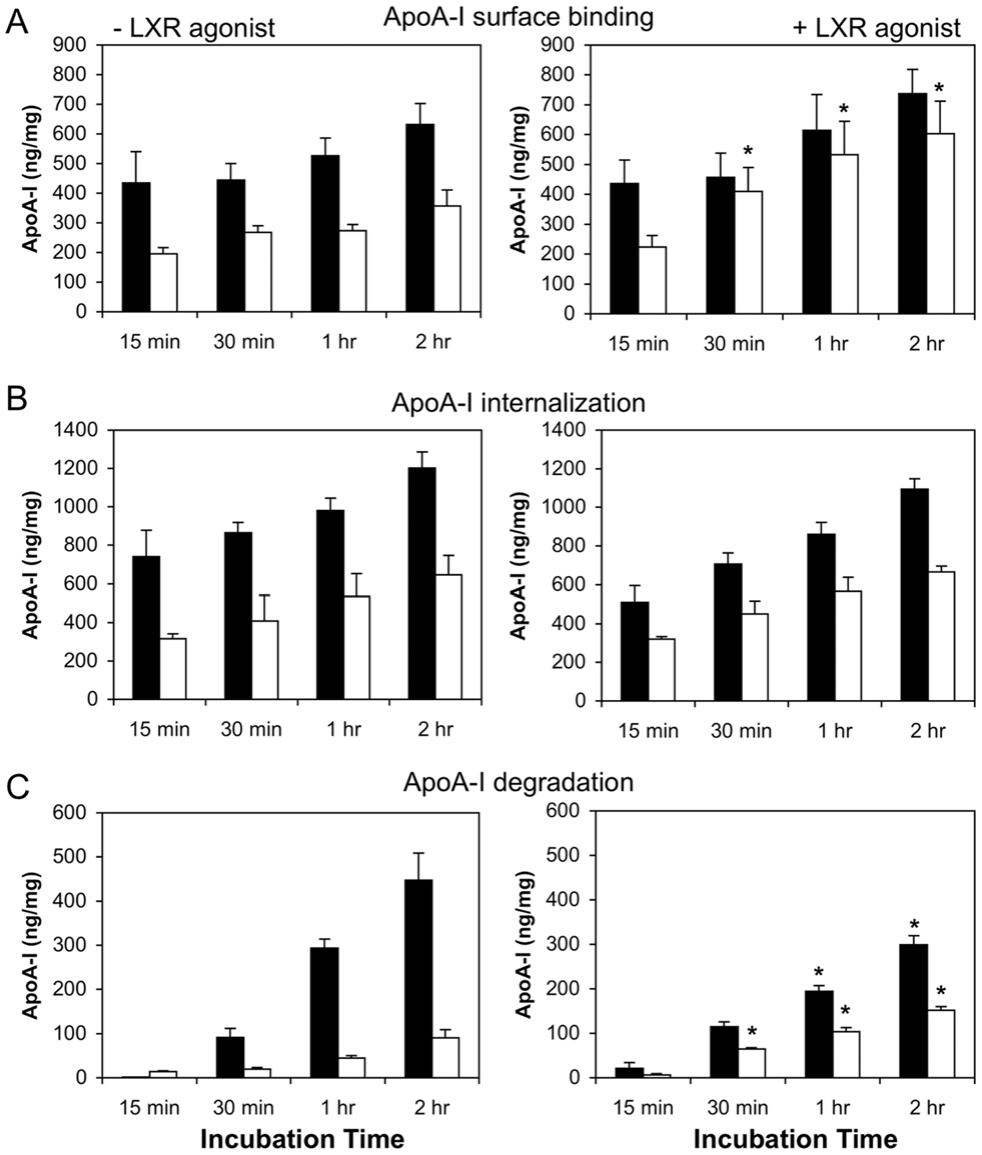
Specific apoA-I binding (A), internalization (B) and degradation (C) in WT and Cav1^**−/−**^ MEFs at 37°C without (left column) and with (right column) prior LXR agonist T0901317 treatment. WT (filled bars) and Cav1^−/−^ (hollow bars) MEFs were incubated with 25 µg/mL ^125^I-apoA-I ±10-fold unlabeled excess at 37°C for 15 min to 2 h. Media were collected and cells were incubated with 0.5 mg/mL trypsin for 30 min at 4°C on ice. Supernatants were collected and counted to determine surface-bound apoA-I. Cells were lysed and ^125^I-apoA-I counted to determined internalized apoA-I without surface-bound and degraded apoA-I. Degraded apoA-I in the medium was determined as TCA-soluble ^125^I-apoA-I. In A–C, specific apoA-I levels are shown with non-specific apoA-I subtracted. Total cell-associated apoA-I is the sum of surface bound (A) and internalized (B) apoA-I. ApoA-I levels are expressed relative to cell protein. Data are average ± standard deviation of one of two representative experiments performed in triplicate cell cultures. Asterisks indicate a significant difference (P>0.05) ± LXR agonist treatment.

We investigated the binding, uptake and degradation of ^125^I-labeled apoA-I by WT and Cav1^−/−^ cells at 37°C (Fig. 4). As with apoA-I binding at 4°C (Fig. 2), apoA-I binding at 37°C was markedly reduced in Cav1^−/−^ MEFs compared to WT MEFs at all incubation times (Fig. 4A, left panels). ApoA-I internalization (Fig. 4B, left panel) was also significantly lower in Cav1^−/−^ MEFs. This is in agreement with a previous report in which apoA-I surface binding strongly correlates with apoA-I internalized over a range of different cell conditions [42]. Degradation of apoA-I (Fig. 4C) could only reliably detected from 30 min onwards, when WT cells degraded apoA-I 5-fold faster that Cav1^−/−^ MEFs. This is also in agreement with previous findings that suggest that internalized apoA-I after a 2 h incubation at 37°C is largely targeted for lysosomal degradation [44]. Hassan et al. did not find any significant degradation of apoA-I in ABCA1-overexpressing BHK cells [42] while apoA-I endocytosed by macrophages is largely degraded [43] suggesting a cell-type specific effect.

ApoA-I and ABCA1 colocalize in endosomal compartments [61], [62], [63] and recent conflicting reports have implicated [41], [42] or refuted [43], [44] the role of ABCA1 in apoA-I internalization, degradation and re-secretion. Here we assessed the effect of ABCA1 upregulation on apoA-I processing in WT and Cav1^−/−^ MEFs. Interestingly, when ABCA1 expression was upregulated by T0901317 (Fig. 4 right panels), apoA-I binding at 37°C was initially the same as non-induced cells but increased up to 1.7-fold and 2.7-fold on WT and Cav1^−/−^ MEFs, respectively, after 2 h incubation with apoA-I. It has been previously suggested that apoA-I stimulated membrane reorganization at the cell surface at 37°C [54], [64] in an ABCA1-dependent manner [65] to create ‘mushroom-like’ membrane protrusion to which apoA-I binding preferentially. Hence the increase in apoA-I binding sites in Cav1^−/−^ MEFs may be apoA-1-induced, ABCA1-dependent membrane protrusions.

Although consistently lower than in non-treated cells (left panel), treatment with the LXR agonist did not significantly alter apoA-I internalization in either cell type (Fig. 4B, right panel) so that WT cells displayed higher apoA-I internalization compared to Cav1^−/−^ MEFs. ApoA-I degradation after 1 h at 37°C was lower upon LXR agonist treatment of WT MEFs but higher upon treatment of Cav1^−/−^ MEF (Fig. 4C, right panel). Hence LXR agonist treatment may result in retaining apoA-I at the cell surface in WT MEFs with a concomitant decrease in internalization and degradation while in Cav1^−/−^ MEFs, LXR agonist treatment in conjunction with apoA-I incubation increases apoA-I binding and degradation.

Taken together the data suggest that expression of caveolin stimulates apoA-I surface binding, uptake and degradation in fibroblasts. In contrast, ABCA1 expression alone (e.g. short apoA-I incubation or low temperatures) does not increase apoA-I binding sites in WT or Cav1^−/−^ MEFs. Since apoA-I degradation is severely reduced in Cav1^−/−^ MEFs, our data suggest that caveolae-mediated uptake of apoA-I leads to degradation.

### Cholesterol efflux from WT and Cav1^−/−^ MEFs

**Figure 5:**
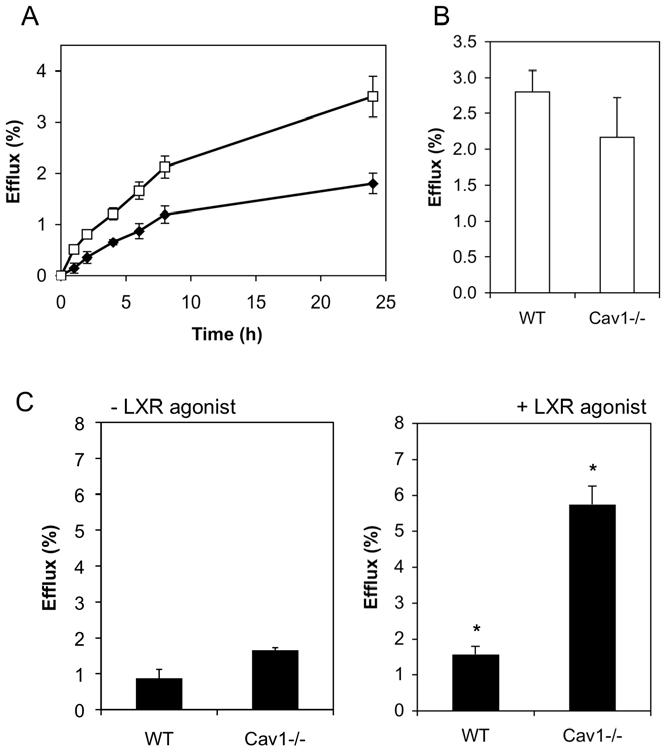
ApoA-I-induced cholesterol efflux from WT and Cav1^**−/−**^ MEFs. **A.** Kinetics of cholesterol efflux to 10 µg/mL apoA-I from WT (closed diamonds) and Cav1^−/−^ MEFs (open squares) over 24 h. Data is one representative experiment performed in triplicate cell cultures. **B.** Phospholipid efflux to apoA-I from WT and Cav1^−/−^ MEFs after 24 h. **C.** Cholesterol efflux (6 h) to 10 µg/mL apoA-I from WT (0.5±0.1 nmol/mg cell protein) and Cav1^−/−^ MEFs (1.0±0.1 nmol/mg) in the absence (left panel) and following treatment with the LXR agonist T0901317 (right panel; WT MEF: 0.9±0.2 nmol/mg; Cav1^−/−^ MEFs: 3.7±0.6 nmol/mg) was determined as described in *Methods*. The data is one representative experiment of six, each performed in triplicate cell cultures. Asterisks indicate a significant difference (P>0.05) ± LXR agonist treatment.

Previous reports have suggested that apoA-I or HDL uptake and recycling back to the plasma membrane facilitates cholesterol export [40], [61], [66]. This debate has become more intense with the conflicting data on the role of ABCA1 in this retrograde cholesterol transport route [41], [42], [43], [44]. Given the different internalization rates of apoA-I in WT and Cav1^−/−^ MEFs, we analyzed cholesterol efflux to apoA-I and HDL from these cells (Fig. 5). Consistent with previous findings [10], we observed that loss of Cav1 expression had no effect on cholesterol efflux stimulated by HDL (data not shown). Efflux to HDL depends on SR-BI and ABCG1 those levels are similar in both cell types (Fig. 1B and data not shown). We also found no difference in total cholesterol levels or unesterified cholesterol between these two cell types, although cholesterol esters were elevated in Cav1-deficient cells (Table 1). However, we did detect a consistent and reproducible 1.7 (±0.5)-fold increase in cholesterol efflux to apoA-I from Cav1^−/−^ MEFs compared to WT MEFs over a 24-hour efflux period (Fig. 5A). In contrast, phospholipid efflux to apoA-I was comparable between the two cell types (Fig. 5B, P>0.05). It should be noted that much lower apoA-I concentrations are required to saturate cholesterol efflux (∼10 µg/mL) than apoA-I surface binding (∼50 µg/mL, Fig. 2A) suggesting that only a small proportion of surface-bound apoA-I functions in cholesterol efflux. In summary, WT MEFs have more apoA-I binding sites and higher rates of apoA-I internalization but lower cholesterol efflux than Cav1^−/−^ MEFs. Hence the data show no correlation between internalization and cholesterol efflux and indicates that an efficient efflux pathway from caveolin-deficient cells exists, which is unlikely to require apoA-I uptake.

**Table 1:** Cholesterol (Chol) content of WT and Cav1^−/−^ MEFs.

	Total Chol.nmol/mg	Free Chol.nmol/mg	Esterified Chol.nmol/mg
WT MEFs	58.6±4.9	56.3±5.8	2.3±1.9 (4.0%)*
Cav1^−/−^ MEFs	65.8±9.7	58.6±8.3	7.2±2.8 (10.8%)

Unesterified (free) and esterified cholesterol was analyzed by HPLC and normalized to cell protein. Values are mean (± S.D.) of six separate cultures. Values in brackets give the percentage of esterified cholesterol of total cholesterol. Asterisk indicates a significant difference of P<0.05.

We next examined cholesterol efflux from cells treated with the LXR agonist. This treatment had no effect on cholesterol efflux to HDL (data not shown) but increased apoA-I-dependent efflux 2.0-fold and 3.3-fold from WT and Cav1^−/−^ MEFs, respectively (Fig. 5C). Thus increasing ABCA1 expression increased cholesterol efflux from both cell types. Curiously, efflux from Cav1^−/−^ MEFs is higher than from WT MEFs despite the lower ABCA1 surface expression in this cell type (Fig. 1D). It has previously been suggested that the rate-limiting step in cholesterol efflux to apoA-I is the intracellular cholesterol transport [49]. In MEFs, LXR agonist treatment disproportionally increased intracellular ABCA1 (compare Fig. 1B to Fig. 1D) and thus is possible that T0901317 treatment enhances efflux from MEF by stimulated intracellular cholesterol trafficking, as previously suggested [61]. Taken together, our data suggest that a surface pool of apoA-I outside caveolae mediates ABCA1-dependent efflux, for which Cav1-mediated apoA-I internalization may not be required.

## Discussion

**Figure 6:**
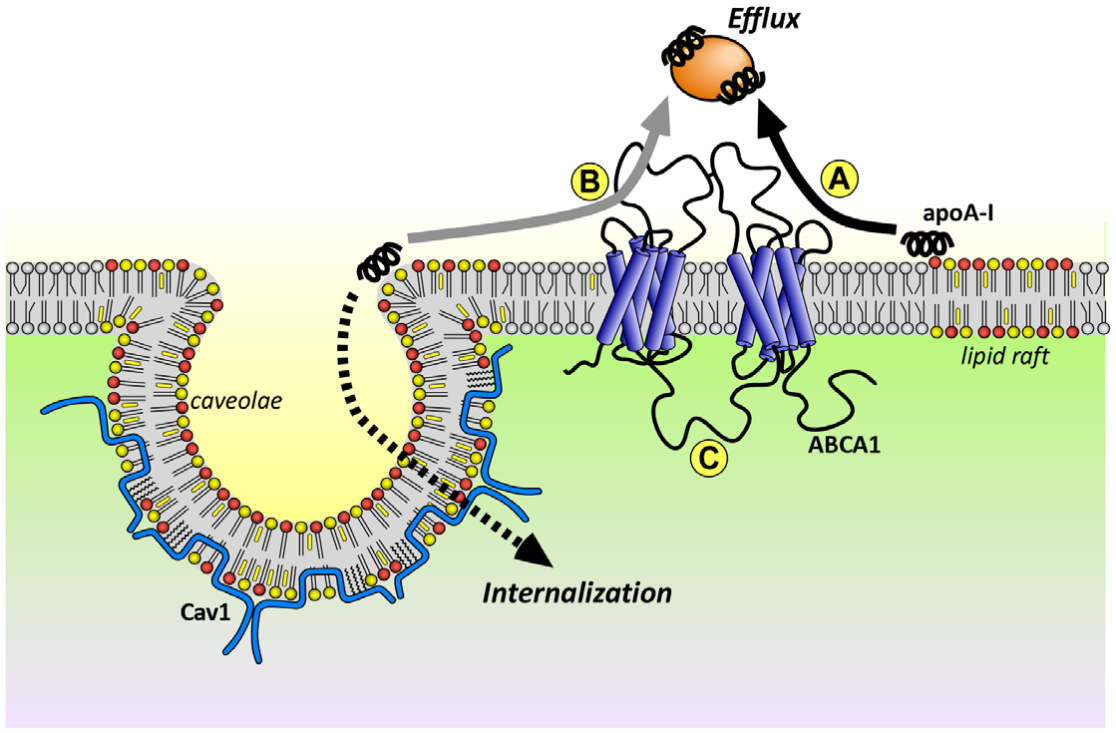
Model of the role of membrane domains in apoA-I-mediated cholesterol efflux. Predominantly saturated phospholipids (color red and yellow) and cholesterol (yellow squares) form domains (lipid rafts, caveolae) that are distinct from more fluid, non-raft domains (grey lipids). **A.** High-capacity apoA-I binding sites preferentially form at the raft/non-raft border where disordered lipid packing creates space for the insertion of apoA-I's amphipathic α-helices between the phospholipids polar head groups. **B.** Caveolae at the plasma membrane form a ring of high membrane curvature at its neck to which apoA-I may bind with high capacity. ApoA-I bound at the caveolae neck is targeted for internalization and degradation (dashed arrow) and is not required for lipid efflux (grey arrow) **C.** ABCA1 expression does not mediate high capacity apoA-I binding sites. Stimulation of ABCA1 translocase activity by apoA-I facilitates efflux (solid black arrow) and may create additional apoA-I binding sites.

Caveolae are generally regarded as a subclass of lipid rafts involved in signal transduction [1], membrane trafficking [67] as well as mechanosensing [68] and mitosis [69]. Here we examined the effect of Cav1 expression on apoA-I surface binding, internalization and export using WT and Cav1^−/−^ MEFs. While fibroblasts do not normally accumulate or export large quantities of cholesterol *in vivo*, we nevertheless chose this model cell lines to assess this question because the expression of transports such as ABCA1, ABCG1 and SR-BI, the level of membrane-associated cholesterol and abundance of lipid rafts are all similar in both cell types under basal conditions. The underlying mechanisms of apoA-I processing and cholesterol efflux should be cell-type independent but are controlled by the expression levels of lipid transporters. Similarly, while quantitative differences exist between that rates of cholesterol efflux between mouse and human cells, the processes are qualitatively similar, with cholesterol efflux to apoA-I being primarily dependent on ABCA1 in both species. We can draw three main conclusions from this work. Firstly, Cav1 but not ABCA1 expression *per se* increases the number of apoA-I binding sites on the cell surface of MEFs. Secondly, Cav1-mediated apoA-I binding enhances apoA-I internalization and degradation. Thirdly, Cav1-mediated apoA-I binding and uptake does not correlate with cholesterol export. In summary, our data suggest that non-caveolar domains provide the surface pool of apoA-I that functions in cholesterol efflux in fibroblasts while caveolae act as entry portal targeting apoA-I for degradation (Fig. 6). These two apoA-I processing pathways are likely to function independently.

The contradicting reports of the role of Cav1 in cholesterol export could be due to the different cell types used in these studies [10], [28], [29], [31], [32], [33], [34], [37], [38] that exhibit various expression levels of lipid transporters. Further, knock-down of Cav1 expression, used in [31], [32], [33], may not completely abolish caveolae from the cell surface, while caveolin over-expression, employed in [28], [29], [34], [38] may not enhance caveolae formation since expression levels of cavins can become limiting factor in caveolae generation [3]. In addition, manipulating Cav1 expression level can affect cholesterol levels and the intracellular cholesterol balance, expression and distribution of lipid transporters such as ABCA1, which may alter cholesterol distribution in the plasma membrane [19], [20], [21], [22], [55]. All of these effects would result in modulated cholesterol export. In addition to function in mechanosensing [68] and mitosis [69], a number of reports have suggested that caveolae act as entry portals for bacterial toxins and viruses [1], [5]. We found that expression of caveolin correlated with the increased uptake of apoA-I. Furthermore, Cav1-dependent apoA-I uptake enhances apoA-I degradation. The reduced uptake of apoA-I, but enhanced apoA-I-dependent cholesterol efflux in Cav1^−/−^ MEFs suggests that this apoA-I uptake pathway is not required for, and possibly competes with cholesterol efflux. At present, we cannot exclude the possibility that a small, caveolin-independent proportion of apoA-I uptake is indeed required for cholesterol efflux from MEFs.

A number of groups investigated whether ABCA1 facilitates apoA-I internalization and whether an ABCA1-dependent retro-endocytosis pathway is a major efflux mechanism. Retro-endocytosis requires the internalization by endocytic carriers and re-secretion of intact, lipidated apoA-I [40], [70] as previously demonstrated by the transcytosis of apoA-I across endothelial cells [71]. ABCA1 itself is internalized during cholesterol efflux [22], [72] but pharmacological retention of ABCA1 on the cell surface correlated with both decreased [41], [73] and increased [73] cholesterol efflux. Hassan et al. showed that apoA-I induced ABCA1 endocytosis to facilitate apoA-I re-secretion [42] in a BHK cell system that over-expressed ABCA1 [55]. In contrast, Denis et al. and Faulkner et al. found that the majority of internalized apoA-I was targeted for degradation in macrophages and fibroblasts with the minor amount of re-secreted apoA-I being insufficient to qualify as a major cholesterol export pathway [43], [44]. Furthermore, inhibition of apoA-I internalization did not decrease cholesterol efflux [44] and CHO cells transfected with ABCA1 displayed enhanced efflux but decreased apoA-I uptake [43]. It should be noted that caveolin expression was not independently assessed in these studies and that the cell types used have vastly different levels of caveolae. Hence the basal condition i.e. no or little ABCA1 expression may not be comparable in these reports. The latter reports in fibroblasts and macrophages are consistent with our data that suggest that Cav1-dependent but ABCA1-independent endocytosis mediates apoA-I degradation and is not required for cholesterol efflux from embryonic fibroblasts.

We found a striking reduction in the number of apoA-I binding sites on the cell surface on Cav1^−/−^ compared to WT MEFs. Surprising the additional apoA-I binding sites in WT cells were not found in the raft fraction where Cav1 elutes but in the non-raft fraction. It should be kept in mind that the fractionation method is not a definite proof of raft association. However the result does suggest that apoA-I association with caveolae is rather weak or that apoA-I binds to the caveolae/plasma membrane interface (see below). Irrespectively of the precise location of the Cav1-mediated apoA-I binding sites on the cell surface, it is unlikely that these binding sites are required for cholesterol efflux. In this context it should be pointed out that relatively low concentrations of apoA-I (10 µg/mL) are sufficient to achieve maximum cholesterol efflux while apoA-I binding to the cell surfaces saturates at much higher concentrations (>50 µg/mL). Hence, few specific apoA-I binding sites may efficiently facilitate cholesterol efflux. Since the apoA-I binding affinity to the surface of Cav1^−/−^ and WT MEFs is similar, our data proposes that such specific, high-affinity binding sites are independent of Cav1 expression.

Phillips and co-workers [64] proposed an efflux model with two distinct types of apoA-I-membrane interactions: (i) apoA-I binding to ABCA1 occurs at low capacity (∼10% of total apoA-I binding) but is critical for the activation of the lipid translocase and (ii) apoA-I binding directly to lipids at a high capacity, which mediates lipid removal from the plasma membrane. For the latter, perturbed phospholipid bilayers [74] or membranes with high curvatures are preferred since the extra space between the polar head groups of the phospholipids allows the insertion of the amphipathic α-helices of apoA-I. The insertion the C-terminal α-helix of apoA-I into the cell membranes and protein-free lipid bilayers is critical for the extraction of lipids, as demonstrated previously with apoA-I truncation mutants [74]. The seemingly contradicting finding of Cav1-induced apoA-I binding sites that have the biochemical characteristics of non-raft domains can be reconciled if apoA-I binds to the neck of caveolae. The high curvature at the caveolae neck makes the membrane packing unfavorable for raft lipids and thus may constitute domains that preferentially partition into non-raft fractions.

Based on the mechanisms described above [64], we propose a model (in which apoA-I binds specifically to domain interfaces, such as the interface of raft and non-raft domains or the necks of caveolae (Fig. 6). This is consistent with our observation that Cav1 expression increases the number of apoA-I binding sites but not the proportion of apoA-I associated with raft domains. Further, increased expression of ABCA1 would have little effect on the overall number of initial apoA-I binding sites in both WT and Cav1^−/−^ cells, as previously reported for macrophages [54], since ABCA-1 is unlikely to increase the domain interface. ApoA-I interaction with domain interfaces would explain why cholesterol export is dependent on an initial interaction of apoA-I with lipid raft domains in the plasma membrane [47] although ABCA1 and ABCA1-dependent cholesterol efflux is not raft-associated [18]. Vedhachalam et al. further suggest that ABCA1 translocase activity forces the membrane to bend inducing filopodia formation and thus additional apoA-I binding sites [64]. This hypothesis could explain our observation of increased apoA-I binding sites on Cav1^−/−^ cells with high ABCA1 expression upon prolonged incubation at 37°C. This was not observed in WT cells where these additional sites may be obscured by caveolae-mediated binding sites. As previously suggested [64], in our model ABCA1 mainly functions as a lipid translocase to aid the lipid extraction process of apoA-I already bound to the plasma membrane. Since cholesterol efflux saturates at relatively low apoA-I concentration, Cav1-independent, high affinity apoA-I binding sites, rather than ABCA1 expression levels at the surface, may be rate limiting for cholesterol efflux to apoA-I.

In conclusion, our study illustrates the usefulness of Cav1-deficient fibroblasts as an experimental model that allows the delineation of Cav1- and ABCA1-controlled apoA-I pathways. While caveolae may constitute binding sites and entry portals for apoA-I, it is unlikely that these high capacity binding sites and Cav1-mediated internalization of apoA-I is required for efflux. Instead, our data suggest that membrane domains other than caveolae compensate for the loss of apoA-I binding in Cav1^−/−^ cells and imply that lipidation of apoA-I takes place at the cell surface in a process that is ABCA1-dependent. It has been demonstrated that ABCA1 resides in non-raft domains [22], [47] but the precious location of apoA-I lipidation is yet to be resolved. One possibility is that raft domains or domain interfaces in the vicinity of ABCA1 provide the reservoir of substrate for ABCA1. Identification of the preferred substrate of ABCA1 may elucidate the interdependencies of apoA-I, lipid domains and ABCA1 that are necessary to facilitate efflux.
